# Neural mechanisms of cognitive conflict: processing COVID-19 vaccine misinformation

**DOI:** 10.3389/fnins.2025.1661523

**Published:** 2026-01-19

**Authors:** Morgan Chase McClellan, C. Brock Kirwan, Stefania R. Ashby

**Affiliations:** 1 Center for Neuroscience, Brigham Young University, Provo, UT, United States; 2 MindCORE, University of Pennsylvania, Philadelphia, PA, United States; 3 Department of Psychology, Brigham Young University, Provo, UT, United States

**Keywords:** decision-making, functional MRI (fMRI), misinformation, prior attitudes, semantic memory, vaccine hesitancy

## Abstract

**Introduction:**

The rapid spread of misinformation during the COVID-19 pandemic has raised important questions about how individuals evaluate false information, particularly when it conflicts with strongly held prior attitudes. Understanding the neural mechanisms underlying the processing of vaccine-related misinformation may clarify why such beliefs persist despite corrective information.

**Methods:**

Using functional magnetic resonance imaging (fMRI), we examined neural responses to COVID-19 vaccine misinformation and factual information in vaccine-receptive participants (*N* = 29). During scanning, participants were presented with a series of vaccine-related statements—both accurate and false—and indicated their level of agreement. Analyses focused on the influence of prior beliefs and attitude congruence on neural activation.

**Results:**

Contrary to our hypotheses, exposure to attitude-incongruent misinformation did not elicit significant activation in emotional processing regions such as the amygdala or precuneus. However, when participants made attitude-incongruent responses—endorsing misinformation or rejecting accurate information—we observed increased activation in the left dorsolateral prefrontal cortex (DLPFC), dorsomedial prefrontal cortex (DMPFC), intraparietal sulcus (IPS), and middle frontal gyrus (MFG), regions associated with decision-making, moral reasoning, memory integration, and cognitive control.

**Discussion:**

These findings suggest that conflicts between incoming information and prior attitudes engage effortful, higher-order cognitive systems rather than affective processing. Resolving such conflicts appears to rely on decision-making and control mechanisms that manage uncertainty and cognitive dissonance. Due to recruitment limitations, the present study focused exclusively on vaccine-receptive individuals. Future research should investigate whether vaccine-resistant individuals exhibit similar or distinct neural patterns, which may provide further insight into the persistence of misinformation in the context of negative prior attitudes.

## Introduction

In 2020, the SARS-CoV-2 virus, which caused coronavirus disease 2019 (COVID-19), precipitated a global health crisis that had profound societal impacts (Ghebreyesus, 2020). Though the impact of the virus rocked health care systems and lead to the deaths of many (Kadri et al., 2021; Cronin and Evans, 2021) the virus was not the only damaging thing that spread extensively during the crisis. The rapid spread of misinformation regarding the virus’s origins, public health measures, and COVID-19 vaccines also made waves across the internet (Gabarron et al., 2021). This “infodemic,” characterized by an increase in false or misleading information during a disease outbreak (World Health Organization, n.d.; World Health Organization, 2020), had far-reaching consequences that contributed to a decline in public trust in health authorities, increased risk-taking behaviors, and led to widespread vaccine hesitancy (Loomba et al., 2021).

The COVID-19 pandemic also spurred a renewed interest in understanding the psychological effects of exposure to misinformation. According to the American Psychological Association, misinformation is defined as incorrect information that is not necessarily intended to deceive (American Psychological Association, n.d.) but can lead to the formation of false beliefs (Bode and Vraga, 2015). Importantly, past work indicates that exposure to misinformation can cause psychological harm by inducing emotional distress—including fear, cynicism, and doubt (Sharov, 2020; Ahmad and Murad, 2020; Mejia et al., 2020)—which may exacerbate emotional responses to conflicting views. Critically, misinformation has been shown to damage the trust individuals have in experts and professional organizations (Tran et al., 2020; Loomba et al., 2021) and the confusion generated by misinformation can undermine informed decision-making (Tran et al., 2020; Bridgman et al., 2020; Zhou et al., 2021) resulting in challenging life experiences being more difficult to navigate.

Reduced trust in experts and professional organizations has also been identified as a key factor influencing vaccine-related attitudes. Prior work has shown that trust in institutions like governmental health agencies and scientific organizations is a strong predictor of COVID-19 vaccine acceptance (Auchynnikava et al., 2025; Grygarova et al., 2025; Jennings et al., 2023; Krastev et al., 2023) and may contribute more to reducing vaccine hesitancy than interpersonal trust (Auchynnikava et al., 2025). Media preference also plays an important role: individuals who consume traditional forms of news media tend to be less vaccine hesitant than those who rely on online media sources (Jennings et al., 2023). Together, these findings demonstrate that the impact of misinformation cannot be understood in isolation from the epistemic context in which individuals form their beliefs. Trust in institutions provides the foundation for regarding certain sources as reliable, while media ecosystems shape the likelihood of encountering, endorsing, or rejecting misinformation.

Among online media ecosystems, social media platforms are especially important to consider as they have emerged as key sources of information for many. In fact, recent estimates from 2024 indicate that one-third of Americans regularly get their news from platforms like Facebook and YouTube (for recent polling data, see Pew Research Center, 2024a; Pew Research Center, 2024b). Though there is some debate in the literature as to the actual level of exposure to misinformation on social media (for discussion, see Budak et al., 2024), several studies have indicated that social media is fertile ground for misinformation to proliferate. For example, work by Vosoughi et al. (2018) showed that false information is 70% more likely to be reshared on social media than correct information. Additionally, Ceylan et al. (2023) found that the majority of false information shared in their study was shared by the most habitual social media consumers (Ceylan et al., 2023). Together these findings indicate that for some individuals the reward-based mechanism built into social media to obtain likes and other interactions (for review, see Metzler and Garcia, 2024) may accelerate sharing of false news on these platforms.

This is further compounded by social media algorithms that curate content aligned with users’ prior beliefs (Bode and Vraga, 2015). The monetization of social media engagement creates a situation where algorithms are incentivized to prioritize engagement with material over accuracy which often results in amplification of sensational or misleading content (i.e., “click-bait”) to maximize revenue (for review, see Brady et al., 2023). Thus, social media feeds frequently reinforce users’ prior biases and decreases the likelihood of encountering corrective information (Cinelli et al., 2021; Del Vicario et al., 2016), laying the perfect foundation for misinformation to take root in memory and influence decision-making. This can have dire consequences, as individuals may make decisions based on misinformation that range from benign misconceptions, such as adding black pepper to food to treat a COVID-19 infection (World Health Organization, 2022), to dangerous errors of judgment, like gargling with a diluted bleach solution or inhaling cleaner fumes to treat a COVID-19 infection (Gharpure et al., 2020; Pennycook et al., 2020).

Though the harms of widespread misinformation propagated on social media are well-known, removing all misinformation from access is neither practical nor possible. Therefore, recent work has shifted towards better understanding the cognitive mechanisms underlying misinformation processing and effective correction strategies (for examples, see Zhang et al., 2022; Efstratiou and De Cristofaro, 2022; Kim and Lim, 2022; Lewandowsky et al., 2012). A growing body of research has shown that once misinformation is learned, it can be challenging to correct, often continuing to influence decision-making even after individuals are presented with correct information (Johnson and Seifert, 1994; Lewandowsky et al., 2012; Susmann and Wegener, 2021). This persistence in memory for the originally learned misinformation may be due to independent storage of misinformation and corrections in memory, leading to difficulties in retrieval, or due to problems with integrating new incoming information with previously learned information during encoding (Lewandowsky et al., 2012; Gordon et al., 2017; Gordon et al., 2019).

Though there is work examining the cognitive mechanisms supporting memory for misinformation and associated corrections, there remains a notable gap in understanding the neural mechanisms that underpin these processes, particularly in the context of prior beliefs. However, it is important to note the distinction between beliefs and attitudes. While related, these constructs reflect different psychological processes: beliefs concern the truth-value of specific propositions (Albarracin and Pitliya, 2022), whereas attitudes reflect an overall evaluative stance on a topic (Ajzen, 2012). Thus, attitudes do not always align perfectly with our item-level beliefs, and in the context of COVID-19 vaccines, individuals may endorse or reject bits of information even when their overall attitudes are clearly vaccine-receptive or vaccine-resistant.

A handful of neuroimaging studies have identified specific brain regions that are activated in response to misinformation, such as the right posterior cingulate cortex, the precuneus, and the amygdala (Gordon et al., 2017; Gordon et al., 2019; Moore et al., 2021). However, to date, results from these studies show little consensus on the neural mechanisms involved in misinformation processing and to our knowledge no studies have investigated how these neural processes are influenced by an individual’s pre-existing attitudes. Given the gaps in our understanding, further investigation of these regions is necessary to provide more insight into the neural mechanisms that contribute to the persistence of misinformation in memory.

In particular, the *amygdala* and *precuneus* are two regions that have remained underexplored but may play a synergistic role in supporting the stickiness of misinformation in memory. The amygdala is well known as a key region for emotional processing (Ball et al., 2009; Patin and Pause, 2015; Said et al., 2009; for review, see Phelps and LeDoux, 2005), but has also been shown to respond robustly to novel and personally significant information (Blackford et al., 2011; Peck et al., 2013; Wang et al., 2018). Given that misinformation often elicits strong feelings (Martel et al., 2020; Luhring et al., 2024)—and this may be especially true when encountering misinformation that conflicts with firmly held attitudes—the amygdala may work to enhance misinformation in memory in these situations (Marsh and Stanley, 2020; Kaplan et al., 2016) by capturing attention and enhancing connections with the hippocampus (McGaugh, 2015).

Once thought of as strictly a visual processing region, the precuneus has been increasingly shown to be involved in episodic memory tasks (Krause et al., 1999; Elman et al., 2013; Foudil and Macaluso, 2024). Recently, it has been proposed that the precuneus may be an important member of two subnetworks of the default mode network (DMN): one that is implicated in supporting episodic memory and another that is engaged during theory of mind or self-referential thinking (for review, see Dadario and Sughrue, 2023). In the context of exposure to misinformation, the precuneus may be a region that is uniquely positioned to support the evaluation of new incoming information in the context of prior memories and pre-existing attitudes. Importantly, the precuneus shares bidirectional structural connections with a variety of other regions implicated in memory and decision-making including the prefrontal cortex, anterior cingulate cortex, and the striatum (for review, see Cavanna and Trimble, 2006). Thus, it may be a region that serves as a mediator working to integrate information from other neural structures—like the amygdala and hippocampus—to support decision-making in light of misinformation.

The purpose of the present study was to investigate the neural mechanisms involved in processing misinformation and factual information regarding COVID-19 vaccines, with a particular focus on how pre-existing attitudes shape these processes. To that end, we exposed groups of COVID-19 vaccine-receptive and vaccine-resistant individuals to COVID-19 vaccine misinformation and factual information while undergoing functional magnetic resonance imaging (fMRI). We hypothesized that regions such as the amygdala and precuneus would exhibit heightened activity when individuals encountered information that contradicted their overall vaccine attitudes, indicating difficulty with integrating new information with previously held attitudes. Furthermore, we predicted that differential neural activation would be influenced by the degree of agreement with both types of information.

## Method

### Public health context during data collection

Data collection for this project was done from the second half of 2022 through approximately June of 2023. By spring of 2023, the United States was estimated to have had over 100,000,000 cumulative cases of COVID-19 (Johns Hopkins, 2023). However, during this time, many were predicting the nearing end of the pandemic (Martín Sánchez et al., 2023), with the Director-General of the World Health Organization (WHO) reporting a hopeful end to COVID-19 as a global health emergency, though still a threat (World Health Organization, 2023a). From 2022 to the end of 2023, the CDC reported a 73.3% decrease in death from COVID-19, likely further suggesting to many people that the end of the COVID-19 pandemic was in sight or reached (Murphy et al., 2024). By the end of 2023, global COVID-19 vaccination rates were estimated to be 67%, with 32% having at least one dose of the booster (World Health Organization, 2023b). In the United States, 82% of the population was estimated to have at least one dose (World Health Organization, 2023b). At Brigham Young University, where these data were collected, COVID-19 vaccination rates were at ceiling due to university vaccination mandates.

### Participants

Forty-three healthy participants were recruited from Brigham Young University via the university SONA research system and from the surrounding community via fliers. Participants received compensation for their participation with their choice of either $20 cash, a ¼-scale 3D-printed model of their structural brain scan, or course credit. Experimental procedures were approved by the university Institutional Review Board and all participants provided written informed consent. Participants were first safety screened for MRI eligibility and verified to be right-handed, fluent in English, and healthy with no history of psychological or neurological conditions or traumatic brain injuries. Four participants were excluded from analyses: one for failing to respond to all conditions in the experiment, one for excessive motion due to talking during the scan, and two due to technical difficulties with MRI data collection resulting in poor data quality. The remaining sample of 39 participants are reported in our analyses.

Due to our interest in how vaccine attitudes may interact with how misinformation is processed in the brain; we also assessed our participants for COVID-19 vaccine hesitancy by asking them whether they were for or against receiving the COVID-19 vaccine (see Appendix for survey questions administered). Using their self-reported vaccine hesitancy, not vaccination status, we classified 10 individuals in our sample as COVID-19 vaccine resistant (7 female, 3 male; ages 18–25 years; M_age_ = 20.90, SD_age_ = 2.38; M_edu_ = 13.40, SD_edu_ = 2.67; 20% received COVID-19 vaccines) and the remaining 29 individuals in our sample as COVID-19 vaccine receptive (14 female, 15 male; age 18–27 years; M_age_ = 21.72, SD_age_ = 2.17; M_edu_ = 12.55, SD_edu_ = 1.30; 96% received COVID-19 vaccines). These groupings were determined based on our participants responses to questions about agreement with COVID-19 vaccination, with those who responded strongly agree or moderately agree being grouped as vaccine receptive and those who responded strongly disagree or moderately disagree being grouped as vaccine resistant, rather than their actual vaccination status as vaccination was mandated by the university from which we recruited our participants.

Due to lower participation rates of vaccine-resistant individuals in our study, we were unable to include group as a factor in our statistical analyses of the neural data as our statistical software was unable to accommodate a strongly unbalanced design (see Preprocessing and neuroimaging data analysis). Accordingly, neural analyses reported here are focused on the vaccine-receptive group only; however, pilot behavioral data we collected from the vaccine-resistant group are presented for transparency. Prior research using fMRI to compare brain activation associated with reading misinformation and corrected information (Gordon et al., 2017) indicated a mean difference in parameter estimate of approximately 23.8 (SD = 28.5; estimated from their Figure 1). We used these estimates to determine our sample size using planned one-sample and paired *t*-tests in G*Power (Faul et al., 2009; Faul et al., 2007). Power analysis indicated that for between-group comparisons we would require at least 21 participants per group.

**Figure 1:**
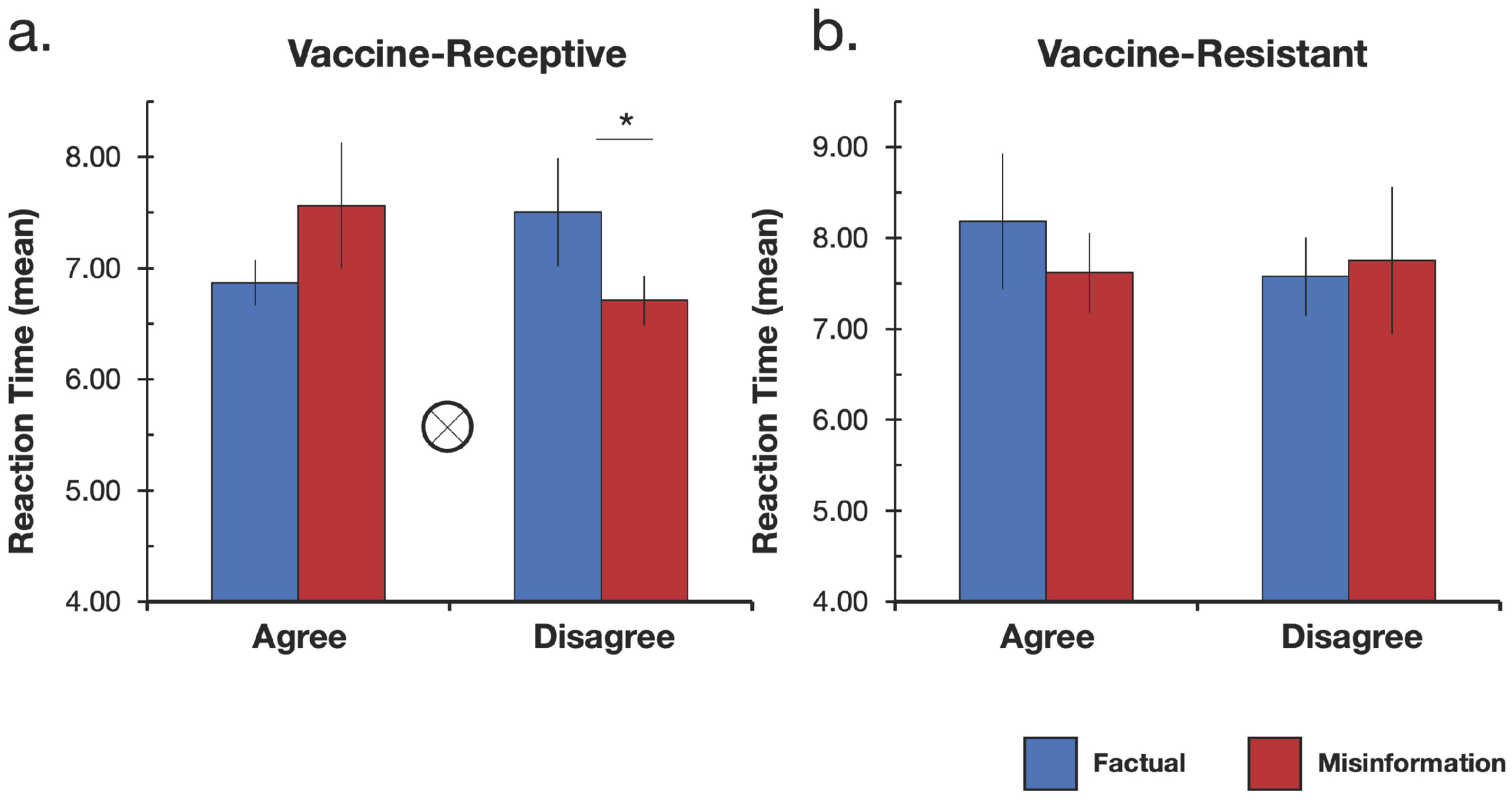
Mean reaction time data for all conditions by group. **(a)** Significant information type (factual vs. misinformation) × endorsement response (agree vs. disagree) interaction for the vaccine-receptive group is denoted by the tensor symbol. Significant interaction was driven by slower reaction times when participants disagreed with factual information compared to when they disagreed with misinformation. **(b)** No significant effects were observed in the vaccine-resistant group. *p < 0.05.

### Stimuli

Stimuli were statements containing either COVID-19 misinformation or COVID-19 facts. Between the fall of 2021 and the spring of 2022, we collected statements from various websites including the Centers for Disease Control (CDC), the Mayo Clinic, NewsGuard Tech, and social media outlets. The research team also generated misinformation statements by crafting statements that directly opposed correct information pulled from reliable sources. In total, we compiled 310 statements (155 categorized as misinformation) as potential stimuli for this study. To select our final pool of statements to be used in the current study and ensure we included statements that would be applicable to both vaccine-receptive and vaccine-resistant participants, we conducted a preliminary pilot study with an independent sample of 44 participants (31 female, 13 male; ages 18–45). In the stimuli pilot study, vaccine-receptive and vaccine-resistant participants rated their level of agreement with various statements. We retained factual statements that were both endorsed by vaccine-receptive participants and rejected by vaccine-resistant participants. Similarly, we retained misinformation statements that were both endorsed by vaccine-resistant participants and rejected by vaccine-receptive participants. Though we did not have a way to verify that participants made truthful ratings rather than socially deisrable ratings, we feel confident that this approach allowed us to narrow down our stimulus pool to statements that would be most relevant for both vaccine-receptive and vaccine-resistant individuals. Using this approach, we reduced our set of statements down to 30 factual and 30 misinformation statements for a total of 60 statements participants viewed while undergoing fMRI in the current study (see Table 1 for example statements, and the OSF link in our open practices statement for a complete list of stimuli).

**Table 1:** A sample of the statements regarding COVID-19 vaccines that were used as stimuli in the current study.

Example statements	Type
The COVID-19 vaccines are unsafe because drug companies created them quickly.	Misinformation
COVID-19 vaccines were developed using science that has been around for decades.	Factual
Getting sick with COVID-19 is the safest and most dependable way to build an immunity to COVID-19, better than getting a vaccine.	Misinformation
Getting a COVID-19 vaccination is a safer and more dependable way to build immunity to COVID-19 than getting sick with COVID-19.	Factual
COVID-19 vaccines are not safe for children and will not protect them or keep them from spreading it at school.	Misinformation
A COVID-19 vaccine can prevent your child from getting COVID-19 and spreading it at home and in school.	Factual
I already had COVID-19, and I recovered, so I do not need to get a COVID-19 vaccine when it is available.	Misinformation
Unvaccinated people who already had COVID-19 are more than twice as likely as fully vaccinated people to get reinfected with COVID-19.	Factual
The Pfizer/BioNTech and Moderna vaccines both contain preservatives.	Misinformation
All COVID-19 vaccines are manufactured with as few ingredients as possible.	Factual

A total of 60 statements (30 misinformation and 30 factual) were shown to participants. A selection of 10 are shown here.

Statements used in the present study ranged from a length of six to 51 words. To verify that the stimuli in our two conditions were equated for word length we conducted an independent samples *t*-test comparing word lengths between factual statements (M_length_ = 18.27, SD_length_ = 10.79) and misinformation statements (M_length_ = 21.30, SD_length_ = 11.77). There was no significant difference in statement lengths between conditions (*t*(58) = 1.041, *p* = 0.302, *d* = 0.27, *two-tailed*) providing reassurance that any differential effects observed between conditions cannot be merely attributed to differences in processing time needed to read stimuli.

### Procedure

Participants viewed 60 statements regarding COVID-19 vaccination while undergoing fMRI. Participants were unaware that they would be viewing both factual information and misinformation regarding COVID-19 vaccines during the study. On each trial, participants were instructed to read the presented statement and respond with their level of agreement for each statement on a 1–4 Likert scale (1 = Strongly Agree, 2 = Moderately Agree, 3 = Moderately Disagree, 4 = Strongly Disagree). Given the polarizing nature of COVID-19 vaccination, we removed a neutral response option to minimize the possibility that participants would choose it to avoid revealing their actual views. In order to have an adequate number of trials in our conditions of interest for analyses, we binned statements rated as a 1 or 2 into a general “Agree” category and statements rated as a 3 or a 4 into a general “Disagree” category. Statements were presented on the screen using PsychoPy software (Peirce et al., 2019) and remained onscreen until the participant responded with their level of agreement. Maximum presentation time was restricted to 10,000 ms for each statement after which an inter-stimulus-interval fixation cross (500–1,500 ms, *jittered*) appeared on the screen between statement trials. Statements were encountered in three blocks of 20 trials with the order of stimuli randomized within and across blocks. Following MRI scanning, all participants were debriefed about their exposure to misinformation and received a take-home packet containing all the statements they read during the study along with each statement’s classification as either factual or misinformation and relevant sources.

### fMRI data acquisition

Imaging data were collected using a 3 T Siemens Vida scanner at the Brigham Young University MRI Research Facility using a 64-channel head coil. Foam padding was placed around the head to minimize head motion. The scanning session started with a localizer SCOUT sequence followed by a standard high-resolution T1-weighted MPRAGE anatomical image [TR = 2,300 ms; TE = 2.32 ms; flip angle = 8°; matrix size = 256 × 256; 192 contiguous slices; FOV = 240 mm; slice thickness = 0.9 mm; voxel size = 0.9 × 0.9 × 0.9 mm] and a GRE-field mapping sequence to assist with preprocessing [TR = 6,490 ms; TE = 16 ms; flip angle = 120°; matrix size = 64 × 64; FOV = 206 mm]. The scanning session concluded with three functional runs of the misinformation task using a standard echo-planar imaging (EPI) sequence [TR = 2000 ms; TE = 30 ms; flip angle = 66°; matrix size = 94 × 94; 78 contiguous slices; FOV = 192 mm; voxel size = 2.0 × 2.0 × 2.0 mm; SMS factor = 3; number volumes = 211].

### Preprocessing and neuroimaging data analysis

Raw dicom images were converted to NIfTI format using *dcm2niix* (Li et al., 2016). Functional, behavioral, and anatomical data were organized in the Brain Imaging Data Structure (BIDS) format for public dissemination on OpenNeuro (doi: 10.18112/openneuro.ds007115.v1.0.0), and structural scans were defaced for public data sharing using in-house scripts to ensure anonymity of the participants. Functional images were analyzed using AFNI (Cox, 1996) and FSL (Smith et al., 2004). Preprocessing began with correcting for signal fallout using the fieldmap data (*epi_b0_correct.py*) and motion correction estimation using the EPI volume with the lowest outlier values as base (*3dVolReg*). Next, we calculated the coregistration of EPI and anatomical scans (*align_epi_anat.py*) and spatial normalization to a lab-specific template in MNI space (*auto_warp.py*). Motion correction, coregistration, and normalization transformations were concatenated and applied in one step (*3dNwarpApply*) to reduce blurring that occurs with separate transformations (Muncy et al., 2017). Quality assurance checks were performed to detect motion outliers that needed to be scrubbed, and functional data were scaled to a mean of 100 and smoothed using a 5 mm FWHM Gaussian kernel.

Trials from four conditions of interest (factual-agree, factual-disagree, misinformation-agree, misinformation-disagree) were modeled using a general linear model (GLM) which also included nuisance regressors representing the six, standard motion regressors for rotational and translational motion. Events from the conditions of interest were modeled with durations matched to reaction times for each trial (M_duration_ = 7.3 s) convolved with the hemodynamic response function as implemented in AFNI’s *3dDeconvolve* program using the response model option *-dmUBLOCK* resulting in beta weight estimations for each condition of interest in each functional run. Residuals from the single subject regression analysis were used to estimate the smoothness of the functional data. These smoothness estimates were then entered into a Monte Carlo simulation to determine spatial extent thresholds for multiple comparisons correction of the group analyses (Cox et al., 2017). All tests were corrected for multiple comparisons using a voxel-wise threshold of *p* < 0.001 and a spatial extent threshold of 55 voxels, nearest-neighbors level 2 (overall *p* < 0.05).

Though we did attempt to examine neural activation for participants who identified as vaccine-resistant, our analysis did not yield any results due to being severely underpowered (i.e., only 10 subjects with usable data in this group). Furthermore, because of the large imbalance in participants between groups we also could not statistically compare neural activity between our vaccine-resistant and vaccine-receptive groups. Due to the difficulty in recruiting enough vaccine-resistant participants to yield meaningful results, we report results from the vaccine-receptive group only. To examine neural responses to information type (factual vs. misinformation) and endorsement response (agree vs. disagree) in vaccine-receptive participants we conducted a 2 × 2 repeated measures ANOVA with both included as within-subjects factors. Follow-up pairwise comparisons were performed as appropriate when the omnibus ANOVA indicated a significant main effect or interaction.

## Results

### Agreement ratings for factual information and misinformation

We first verified that individuals’ self-identification as either vaccine-receptive or vaccine-resistant matched their responses to the factual information and misinformation presented within our study regarding COVID-19 vaccines. Indeed, we found this to be the case. Vaccine-receptive participants agreed with factual COVID-19 vaccine information and disagreed with misinformation (Table 2). As can be seen from trial counts in Table 2, both groups had very low response rates in the “strongly agree/disagree” bins when the information was inconsistent with prior attitudes. Consequently, for all subsequent analyses we collapsed across strongly/moderately levels, making the agreement responses dichotomous. Although there did appear to be increased variability in responses within the vaccine-resistant group (i.e., much larger standard deviations), this is most likely due to the significantly smaller sample size of this group (*N* = 10) relative to the vaccine-receptive group (*N* = 29). Together, these findings indicate that participants predominately agreed or disagreed with factual information and misinformation in a manner that reflected their self-reported COVID-19 vaccine hesitancy.

**Table 2:** Mean (SD) trial counts across each level of agreement.

Group	Information type	Strongly agree	Moderately agree	Moderately disagree	Strongly disagree
Vaccine-compliant	Correct	15.0 (6.5)	10.6 (5.5)	3.3 (2.4)	0.8 (1.0)
	Misinformation	0.5 (0.7)	3.0 (2.2)	10.4 (6.9)	15.7 (7.8)
Vaccine-hesitant	Correct	0.8 (1.3)	6.9 (6.0)	11.4 (5.7)	10.2 (7.1)
	Misinformation	11.2 (7.6)	11.0 (5.1)	5.8 (6.5)	1.6 (1.6)

There were very low response rates for attitude-incongruent statements. Consequently, analyses presented here collapsed across “strongly” and “moderately” levels. There were 30 trials per stimulus condition (60 total).

### Response times for factual information and misinformation

Next, as an exploratory analysis we examined whether mean response times varied as a function of information type (factual vs. misinformation) and endorsement response (agree vs. disagree). We first conducted a 2 × 2 repeated measures ANOVA with factors for information type (factual, misinformation) and endorsement response (agree, disagree) using all usable participants, both vaccine-receptive and vaccine-resistant. There were no significant main effects of information type (*F*(1, 37) = 0.29, *p* = 0.59, $\eta_{p}^{2}$ = 0.008) or endorsement response (*F*(1, 37) = 0.09, *p* = 0.77, $\eta_{p}^{2}$ = 0.002), and no significant interaction (*F*(1, 37) = 3.07, *p* = 0.09, $\eta_{p}^{2}$ = 0.08) in the overall sample. When considering reaction time data within the vaccine-resistant and vaccine-receptive groups individually there were no significant main effects in either group or a significant interaction within the vaccine-resistant group (Figure 1b). However we did observe a significant interaction between information type and endorsement response within the vaccine-receptive participants (*F*(1, 27) = 5.74, *p* = 0.024, $\eta_{p}^{2}$ = 0.175; see Figure 1a) driven by slower reaction times when vaccine-receptive participants disagreed with factual information compared to when they disagreed with misinformation (*t*(28) = 1.93, *p* = 0.03, *d* = 0.36, *one-tailed*). Together these results indicate that in the overall sample, participants took about the same amount of time to respond to all statements regardless of whether that information was factual or misinformation and regardless of whether they agreed or disagreed with the information. However, vaccine-receptive participants were slower to respond when disagreeing with factual information than when disagreeing with misinformation.

### Neural mechanisms of misinformation processing in the vaccine-receptive group

To look for differences in neural activity as a function of information type (factual vs. misinformation) and endorsement response (agree vs. disagree) within the vaccine-receptive group, we conducted a whole-brain 2 × 2 repeated measures ANOVA. We observed no significant main effects of information type or endorsement response in the brain. However, we did find a significant interaction between information type and endorsement response (*F*(1, 27) = 5.80, *p* < 0.001, $\eta_{p}^{2}$ = 0.643) in four regions (see Table 3): left intraparietal sulcus (IPS), left dorsolateral prefrontal cortex (DLPFC), left dorsomedial prefrontal cortex (DMPFC), and left middle frontal gyrus (MFG).

**Table 3:** Information type (misinformation vs. factual) × endorsement response (agree vs. disagree) interaction for the vaccine-receptive group.

Region	Hemisphere	Cluster size	Peak coordinate		
			*X*	*Y*	*Z*
Intraparietal sulcus (IPS)	L	248	−38	−56	50
Dorsolateral prefrontal cortex (DLPFC)	L	91	−54	22	33
Dorsomedial prefrontal cortex (DMPFC)	L	69	−4	32	54
Middle frontal gyrus (MFG)	L	62	−24	8	68

Significant results here survived corrections for multiple comparisons using a voxel-wise threshold of *p* < 0.001 and a spatial extent threshold of 55 voxels, nearest-neighbors level 2.

**Figure 2:**
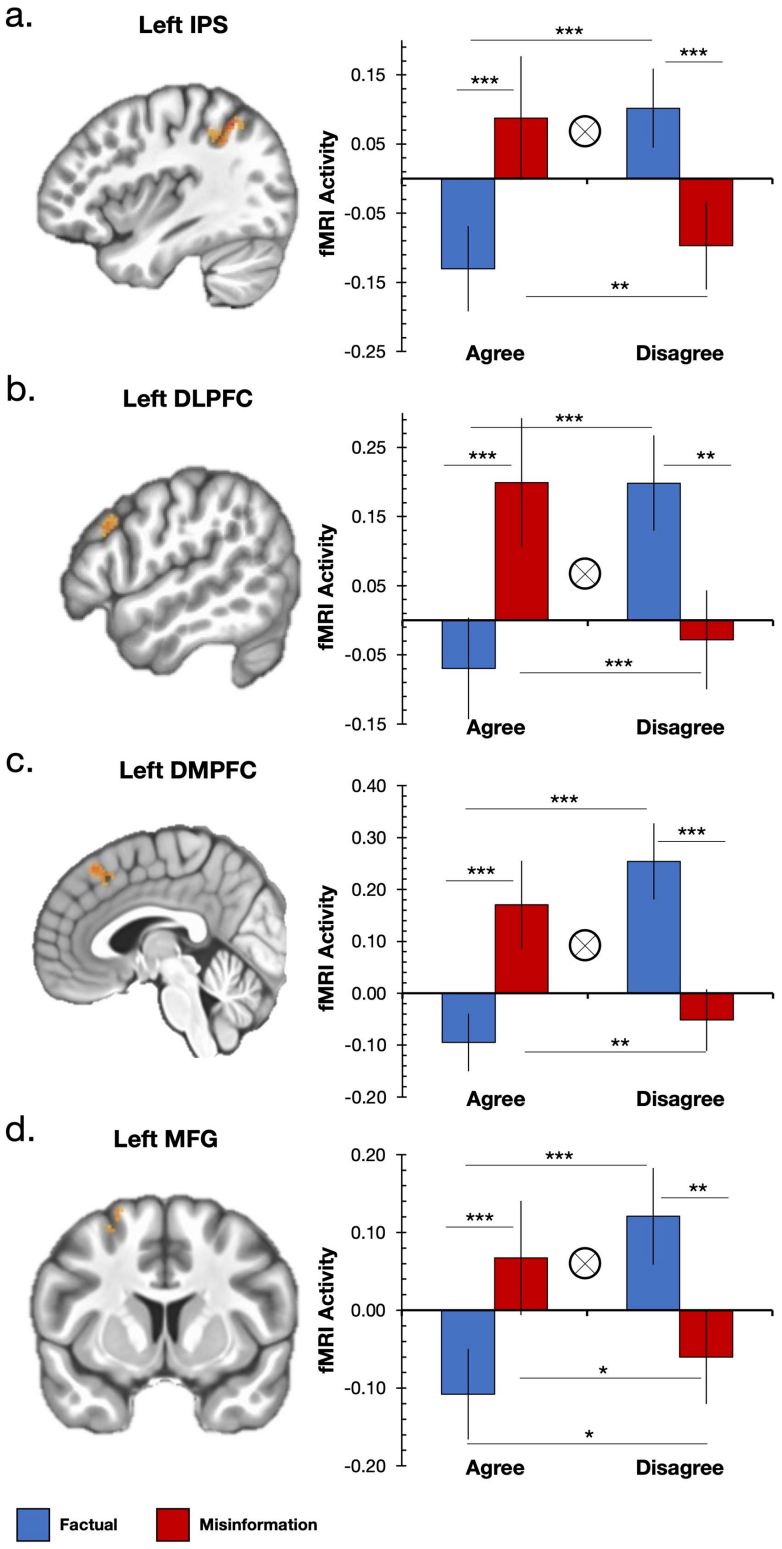
Significant information type (factual vs. misinformation) × endorsement response (agree vs. disagree) interaction for the vaccine-receptive group in four regions: **(a)** left intraparietal sulcus, **(b)** left dorsolateral prefrontal cortex, **(c)** left dorsomedial prefrontal cortex, and **(d)** left middle frontal gyrus. Significant interactions are noted by the tensor symbol and significant pairwise comparisons are noted with asterisks (*p < 0.05; **p < 0.01; ***p < 0.001).

To further characterize the significant interaction in each region we performed follow-up pairwise *t*-tests. Because vaccine hesitancy reflects an overall attitudinal stance rather than endorsement of each specific claim, congruency in the present study is defined based on attitude alignment rather than item-specific beliefs. Overall, we found a pattern of increased activation when participants made attitude-inconsistent judgments, or judgments that conflicted with their pre-existing attitudes regarding COVID-19 vaccines (i.e., endorsing misinformation and rejecting factual information), and decreased activation when participants made attitude-consistent judgments, or judgments that were in line with their pre-existing attitudes regarding COVID-19 vaccines (i.e., endorsing factual information and rejecting misinformation). Indeed, we found that neural activation in the left IPS (Figure 2a) was significantly greater when participants disagreed with factual information than when they agreed with factual information (M_diff_ = 0.23, SD_diff_ = 0.28; *t*(28) = 4.51, *p* < 0.001, *d* = 0.84, *two-tailed*) and significantly greater when participants disagreed with factual information than when they disagreed with misinformation (M_diff_ = 0.20, SD_diff_ = 0.25; *t*(28) = 4.24, *p* < 0.001, *d* = 0.79, *two-tailed*). Left IPS activity was also greater when participants agreed with misinformation than when they agreed with factual information (M_diff_ = 0.21, SD_diff_ = 0.28; *t*(27) = 3.99, *p* < 0.001, *d* = 0.75, *two-tailed*) and greater when participants agreed with misinformation than when they disagreed with misinformation (M_diff_ = 0.18, SD_diff_ = 0.31; *t*(27) = 2.95, *p* = 0.007, *d* = 0.56, *two-tailed*). Similarly significant findings were found for the same relationships within the left DLPFC (Figure 2b) and left DMPFC (Figure 2c).

Additionally, within the left MFG (Figure 2d) we found the same significant relationships as the other regions (all *t*’s > 2.67, all *p*’s < 0.012) but we also found greater neural activity in this region when participants disagreed with misinformation than when they agreed with factual information (M_diff_ = 0.05, SD_diff_ = 0.11; *t*(28) = 2.34, *p* = 0.027, *d* = 0.44, *two-tailed*). Thus, in addition to responding to attitude-inconsistent judgments like the other three regions, the MFG also responded when participants correctly rejected misinformation.

Next, we examined whether the preceding effect (i.e., greater activation for attitude-inconsistent judgments and decreased activation for attitude-consistent judgments) generalized to the vaccine-hesitant group. We examined fMRI activation within the same clusters for an interaction between stimulus type and behavioral response with the hypothesis that the activation pattern would be inverted relative to the vaccine-accepting group. In other words, we expected increased activation when endorsing factual information and rejecting misinformation and decreased activation when endorsing misinformation and rejecting factual information. The interaction was not significant in any cluster (all *p*’s > 0.05), although the numerical difference was in the predicted direction in the left DLPFC, left IPS, and left MFG (see Supplementary Table S1).

Finally, we performed ROI analyses for the bilateral amygdala and precuneus. ROIs were defined from the labels “precuneus” and “amygdala” in the Mindboggle-101 dataset (Klein and Tourville, 2012) warped to our study template using Joint Label Fusion (Wang et al., 2013). Mean betas were extracted from each ROI for every participant and task condition. There were no significant main effects of stimulus type or behavioral response and no interaction for either ROI.

## Discussion

We sought to explore the neural mechanisms underlying the processing of COVID-19 vaccine information, focusing on how pre-existing attitudes about vaccine efficacy and safety interact with the processing of COVID-19 vaccine misinformation. In vaccine-receptive individuals, we found that brain regions associated with executive functioning, decision-making, emotion regulation, and working memory showed increased activation when participants made attitude-inconsistent judgments, such as endorsing misinformation or rejecting factual information. This pattern of neural activity was found exclusively in the left hemisphere and involved key decision-making mechanisms in the prefrontal and parietal cortices.

### Prefrontal engagement during conflicting attitude judgments

The dorsolateral prefrontal cortex (DLPFC) and dorsomedial prefrontal cortex (DMPFC) are regions crucial for decision-making and executive function (Wang et al., 2021; Krain et al., 2006) and recently have been implicated as part of a network of regions responsible for belief processing under uncertainty (Gerchen et al., 2024). The DLPFC, important for integrating new information with existing knowledge (D'Esposito et al., 1999; Christoff et al., 2001; Gold and Shadlen, 2007; Zheng et al., 2024) and working memory processes (Cohen et al., 1997; Fang et al., 2016; Blumenfeld and Ranganath, 2006), may have supported vaccine-receptive participants in retrieving prior beliefs and weighing them against new information for potential integration into long-term memory.

It is also possible that the DLPFC activity observed in our study reflected moral decision-making processes as prior work has indicated that DLPFC activity tracks moral judgments. For example, Buckholtz et al. (2008) found increased activation in the right DLPFC while subjects made responsibility judgments in the context of observing simulated criminal activity during fMRI. Similarly, Pretus et al. (2019) found that an individual’s self-reported willingness to fight and potentially die for a moral cause is mediated by activation in the DLPFC. Thus, in the current study the DLPFC may have helped participants with integrating new incoming information with existing knowledge as well as evaluating the moral implications of that information in light of pre-existing attitudes.

The DMPFC, which plays a role in social judgment (Wittmann et al., 2025; for review, see Gangopadhyay et al., 2021), may support this function by suppressing irrelevant information (Mahmoodi et al., 2023) and regulating emotional responses (Berboth and Morawtz, 2021). In our task, DMPFC activation may reflect managing initial negative reactions to attitude-incongruent information. This region is also implicated in both the encoding and retrieval of emotional memories (Kensinger and Ford, 2021), suggesting it may facilitate the integration of conflicting emotional content with existing memory traces. Thus, DMPFC engagement may have contributed to mitigating affective interference, allowing participants to override pre-existing attitudes and commit attitude-inconsistent judgments in our task.

Together, the DLPFC and DMPFC may function synergistically, with the DLPFC supporting the comparison of new information against pre-existing attitudes and the DMPFC flagging attitude-inconsistent information for further scrutiny. The increased activity we saw in the DMPFC during these kinds of judgments suggests that it may help downregulate the negative emotions that are most likely associated with conflicting information, potentially allowing participants to momentarily override pre-existing attitudes and make judgments that were incongruent with their pre-existing attitudes. Another possibility, however, is that prefrontal engagement reflected not only incongruence between personal attitudes and the presented statements, but also cognitive conflict between participants’ personal attitudes and what they perceived as the expectations of the research setting. Vaccine hesitancy has been a highly polarizing issue, shaped in part by social pressures, and it is possible that being in a university setting—where vaccination was both mandated and publicly encouraged—created an added layer of perceived social desirability. Consistent with this interpretation, a meta-analysis by Wu et al. (2016) demonstrated the prefrontal cortex is engaged when personal judgments conflict with group opinions and is also predictive of social conformity to reduce cognitive dissonance. Thus, part of the neural activity we observed in prefrontal regions may reflect participants reconciling their own attitudes with what they deemed to be the institutionally “correct” or socially desirable stance.

Our neural findings may not only reflect a resolution of cognitive conflict but could also be shaped by the degree of certainty participants felt about their endorsement of the presented information. Although participants reported their level of agreement with each statement—potentially a proxy for measuring certainty or confidence—we did not obtain enough responses in each agreement bin to analyze them as distinct conditions (see Supplementary Figure 1). As a result, we collapsed responses into broader “agree” and “disagree” categories, limiting our ability to assess the degree of certainty in participant’s judgments. Still, it remains possible that the DLPFC and DMPFC activation patterns reflect variations in certainty. For example, Gerchen et al. (2024) found increased activation in the DLPFC when participants indicated high certainty in their responses and increased activation in the DMPFC when participants indicated low certainty in their responses. Future work should further explore the idea that DLPFC and DMPFC work synergistically to manage confidence when incoming information conflicts with pre-existing attitudes during decision-making.

### Attention and response inhibition in uncertain decision-making

Similarly, we observed the same pattern of increased activation during attitude-inconsistent judgments in the left intraparietal sulcus (IPS) and left middle frontal gyrus (MFG). While the IPS is often associated with perceptual-motor coordination (for review, see Culham and Kanwisher, 2001), it is also implicated in voluntary attention (Kincade et al., 2005; Corbetta and Shulman, 2002) and decision-making under uncertainty (Vickery and Jiang, 2009). Thus, in this context, the involvement of the IPS may reflect heightened attentional demands as participants attempted to navigate attitude-incongruent misinformation that potentially provoked uncertainty.

The MFG has historically been active during tasks involving response inhibition where participants are inhibiting a prepotent response (Collette et al., 2001; Collette et al., 2005; for review, see Ridderinkhof et al., 2004) and when participants are actively attempting to suppress a memory (Anderson et al., 2004; Anderson and Hanslmayr, 2014; Benoit and Anderson, 2012). Additional work has also shown that the left MFG is more active when individuals are less confident in their judgments during decision-making tasks (Risius et al., 2013). Consistent with the existing literature, we observed increased MFG activation when vaccine-receptive participants made attitude-inconsistent judgments suggesting participants may have been attempting to suspend their pre-existing attitudes but were also experiencing uncertainty in their responses when they made judgments that were in conflict with their pre-existing attitudes.

Uniquely, we also found greater activation in the MFG when vaccine-receptive participants correctly rejected misinformation compared to when they endorsed factual information. Recent work has demonstrated that temporary lesions in the MFG lead to more rational decision-making but only when participants anticipate negative social consequences (Martin-Luengo et al., 2023). Similarly, a study of the continued influence effect of misinformation found that enhanced activity in the left MFG while learning a retraction (i.e., a correction of previously learned misinformation) predicted more accurate responding at retrieval (Gordon et al., 2017). Thus, our result may be consistent with the idea that this region is sensitive to the social pressure and the cognitive dissonance our vaccine-receptive participants may have felt when encountering attitude-incongruent information. Together, our findings and the broader literature suggest that the MFG not only reflects low confidence during attitude-inconsistent judgments but also evaluates social consequences and internal dissonance when processing information that conflicts with what has previously been learned.

### On the absence of activation differences in key emotion-processing regions

We hypothesized that emotion-processing regions such as the precuneus and amygdala would support decision-making in our task. However, we did not observe activation differences in either region. Prior work has shown the precuneus is engaged when processing retractions of misinformation (Gordon et al., 2017) and in response to emotional valence and information source (Moore et al., 2021). These disparities likely stem from differences in study design. First, our study lacked a retraction condition and did not manipulate information source—which may serve as a reference point during agreement judgments. Our study also did not vary the emotional valence of the information. Although COVID-19 vaccine content is generally emotionally charged, as public discourse and debate regarding the vaccines has been heated, our stimuli may not have elicited strong emotional engagement, potentially limiting precuneus involvement.

More curious was the lack of amygdala activation in the current study. We hypothesized that the information presented in our study would elicit strong emotions and the amygdala would track those emotions. Indeed, past work indicates that misinformation evokes strong emotions ultimately leading to the information being more memorable, believed, or shared (for example, see Luhring et al., 2024). However, work by Edelson et al. (2014) noted that the amygdala engages more robustly during original exposure to misinformation as opposed to during recall. Thus, in the current task it is possible that the misinformation presented throughout was not a first exposure for most of our participants. We selected misinformation from multiple sources and given the high media coverage and social media discussion of COVID-19 vaccines over the years it is possible that we were not measuring neural activity in response to first exposure to misinformation. Thus, future studies should look at the impact of previous exposure to misinformation and the neural mechanisms at play.

### Limitations

The primary limitation of our study was the low participation rate of COVID-19 vaccine resistant volunteers. While the exact cause of this challenge is unclear, we suspect that hesitancy toward science and research participation among this population may have contributed. Sampling was further constrained by the fact that students at our university were required to be vaccinated against COVID-19, which likely biased the available pool of participants or led to individuals who were vaccine-resistant feeling social conformity pressures to align their opinions on vaccination during recruitment with those of the institution where the study was conducted. As a result, we were unable to explore the neural mechanisms within the vaccine-resistant group or test for potential between-group differences. Recruitment difficulties also contributed to variability during pilot testing of our stimuli and to an uneven distribution of agreement and disagreement responses during the fMRI task, which may in turn have influenced the neural activation patterns observed. Additionally, we did not collect information about participants’ previous or planned healthcare occupations, which could have been another source of variability in attitudes toward COVID-19 vaccination.

### Future directions: exploring the neural mechanisms of COVID-19 vaccine resistance

Despite the challenge of recruiting a vaccine-resistant sample, studying this population remains a critical next step. Increasing efforts to engage vaccine-resistant individuals would allow future research to identify similarities and differences in the cognitive and neural underpinnings of attitude processing across a broader population, offering key insights into the persistence of vaccine misinformation in memory. Additionally, future work should further disentangle knowledge and opinion in their studies—for example, including an “I do not understand” option on agreement scales—could help future researchers distinguish between disagreement with a statement and uncertainty about its meaning, offering a clearer picture of whether judgments reflect genuine attitudes or difficulties in comprehension.

In the current study, we asked participants to report their COVID-19 vaccine attitudes before neural data was collected, which may have inadvertently made these attitudes more salient. It is possible that just simply asking participants to reflect on their vaccine-receptivity prior to collecting the neural data could have influenced how they subsequently processed vaccine-related information. Future work should consider examining misinformation processing both when attitudes are not made salient and when they are explicitly activated. Comparing neural responses under these conditions would clarify whether the influence of pre-existing attitudes operates automatically or depends on conscious awareness giving us more insight into how the brain evaluates misinformation in real-world contexts.

Beyond sampling and study design considerations, it will also be important to examine the broader epistemic context in which misinformation is encountered. Prior studies have shown that trust in institutions and media preferences are central predictors of vaccine hesitancy (Auchynnikava et al., 2025; Grygarova et al., 2025; Jennings et al., 2023; Krastev et al., 2023). In the present study, participants were presented with vaccine-related statements without source attribution, and we did not manipulate or measure source credibility directly. Our operationalization of “factual information” was based on consensus among established scientific and public health authorities, while “misinformation” was drawn from false or misleading claims circulating publicly. This approach allowed us to focus on the cognitive and neural processes involved in evaluating content, independent of source. However, individuals do not form attitudes in a vacuum. Given the strong role of institutional trust and media exposure in shaping COVID-19 vaccine attitudes, future research should directly examine how these factors interact with pre-existing attitudes and neural responses to misinformation. Integrating measures with neuroimaging methods could extend the present findings and clarify how real-world contexts interact with the cognitive and neural processing of misinformation.

## Data Availability

The raw data supporting the conclusions of this article will be made available by the authors, without undue reservation. Supplementary information as well as data and materials for all experiments are freely available in the *Neural Mechanisms of Cognitive Conflict* repository on the Open Science Framework (https://osf.io/4b9v8/) and on OpenNeuro.org (doi: 10.18112/openneuro.ds007115.v1.0.0). None of the experiments discussed in the current report were preregistered.

## Appendix

### Survey assessing COVID-19 vaccine attitudes

What is your attitude toward vaccines?

- Strongly Agree with getting vaccinated.
- Moderately Agree with getting vaccinated.
- Moderately Disagree with getting vaccinated.
- Strongly Disagree with getting vaccinated.

What is your attitude toward getting the COVID-19 vaccines?

- Strongly Agree with getting the COVID-19 vaccine.
- Moderately Agree with getting the COVID-19 vaccine.
- Moderately Disagree with getting the COVID-19 vaccine.
- Strongly Disagree with getting the COVID-19 vaccine.

Have you been vaccinated for COVID-19?

- Yes
- No

[If Yes] How has your attitude toward the COVID-19 vaccine changed since you received the vaccine?

- Strongly Agree with getting the COVID-19 vaccine.
- Moderately Agree with getting the COVID-19 vaccine.
- No change
- Moderately Disagree with getting the COVID-19 vaccine.
- Strongly Disagree with getting the COVID-19 vaccine.

[If Yes] How many doses have you received? Please fill out this information using your vaccine card.

- 1
- 2
- 3+

[If Yes] Which vaccines, did you receive? Please fill out this information using your vaccine card *(This question allowed for multiple answers)*.

- Pfizer
- Moderna
- Johnson and Johnson
- Other
  [If Other] Since “Other” was selected, please specify what vaccine you received.
