# Supplementary material for: Neural mechanisms of cognitive conflict: processing COVID-19 vaccine misinformation
Source: Front Neurosci. 2026 Jan 19;19:1661523. doi: 10.3389/fnins.2025.1661523 (PMC12862833; doi:10.3389/fnins.2025.1661523)
Supplement: Supplementary file 1 [file Data_Sheet_1.docx]

**Supplemental Material**

**Supplemental Figure 1.** Proportion of responses by group and information type. **A.** For the vaccine-receptive group, the proportion of responses to each of the four Likert scale ratings split by factual information in blue and misinformation in red. **B**. For the vaccine-resistant group, the proportion of responses to each of the four Likert scale ratings split by factual information in blue and misinformation in red. Proportion of responses for both groups is highly skewed to the upper ends of the scales.


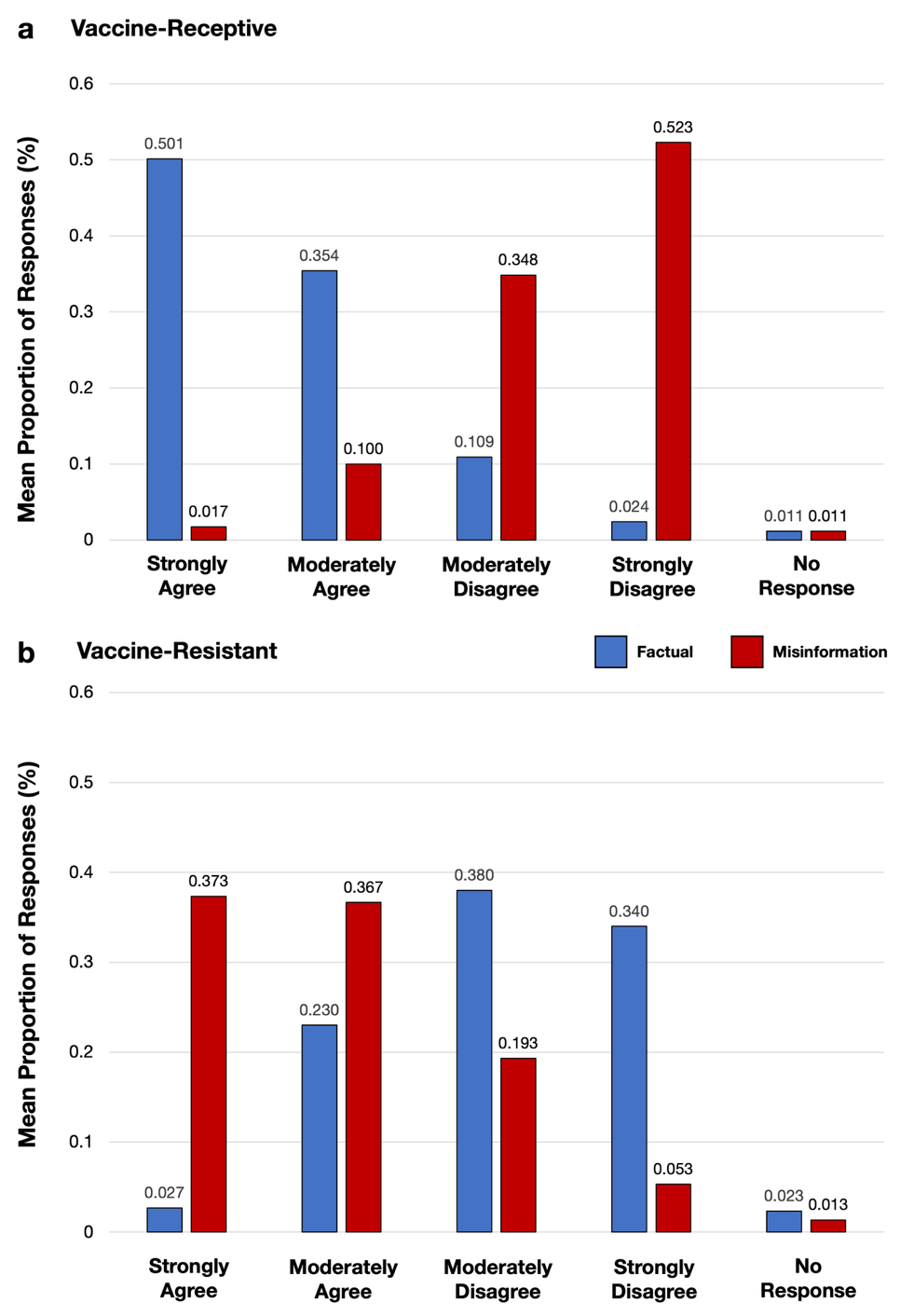


**Supplemental Table S1.** Mean (±SD) beta values for vaccine-hesitant group in regions identified in the stimulus type by response interaction.

|  |  | **Factual** | | **Misinformation** | |
| --- | --- | --- | --- | --- | --- |
| **ROI** | **Group** | **Agree** | **Disagree** | **Agree** | **Disagree** |
| LDLPFC | Vaccine-receptive | -0.0694 ± 0.395 | 0.198 ± 0.371 | 0.187 ± 0.489 | -0.0232 ± 0.385 |
|  | Vaccine-hesitant | -0.0389 ± 0.14 | -0.0441 ± 0.249 | -0.0545 ± 0.35 | 0.0469 ± 0.385 |
| LDMPFC | Vaccine-receptive | -0.0949 ± 0.3 | 0.254 ± 0.395 | 0.159 ± 0.444 | -0.0457 ± 0.319 |
|  | Vaccine-hesitant | -0.0138 ± 0.248 | 0.0785 ± 0.336 | 0.0619 ± 0.416 | 0.212 ± 0.43 |
| LIPS | Vaccine-receptive | -0.13 ± 0.332 | 0.102 ± 0.308 | 0.0721 ± 0.473 | -0.0845 ± 0.338 |
|  | Vaccine-hesitant | -0.0489 ± 0.201 | -0.104 ± 0.206 | -0.126 ± 0.177 | -0.021 ± 0.289 |
| LMFG | Vaccine-receptive | -0.108 ± 0.313 | 0.121 ± 0.334 | 0.0588 ± 0.384 | -0.0542 ± 0.323 |
|  | Vaccine-hesitant | -0.0746 ± 0.186 | -0.0387 ± 0.153 | -0.0651 ± 0.207 | 0.0608 ± 0.265 |
